# Supplementary material for: Woeseiales transcriptional response to shallow burial in Arctic fjord surface sediment
Source: PLoS One. 2020 Aug 27;15(8):e0234839. doi: 10.1371/journal.pone.0234839 (PMC7451513; doi:10.1371/journal.pone.0234839)
Supplement: S1 Table — Completeness and contamination were determined for each genome with CheckM. (DOCX) [file pone.0234839.s002.docx]

S1 Table. Genome statistics for the Woeseiales MAGs in this study. Completeness and contamination were determined for each genome with CheckM.

| MAG | Accession | Size (mbp) | No. contigs | Completeness (%) | Contamination (%) | GC content (%) |
| --- | --- | --- | --- | --- | --- | --- |
| Woeseia sp. stnAB | IMG [2802428844](http://img.jgi.doe.gov/genome.php?id=2802428844) | 2.7 | 388 | 83 | 6 | 57.8 |
| Woeseia sp. stnAC | IMG [255802428845](http://img.jgi.doe.gov/genome.php?id=2802428845) | 3.1 | 331 | 80 | 9 | 55.3 |
| Woeseia sp. stnAC-2 | IMG [2802428847](http://img.jgi.doe.gov/genome.php?id=2802428847) | 4.2 | 684 | 80 | 4 | 55.1 |
| Woeseia sp. stnF | IMG [2802428846](http://img.jgi.doe.gov/genome.php?id=2802428846) | 2.4 | 421 | 94 | 7 | 54.3 |
| Woeseia2 ps. stnF-2 | IMG [2802428848](http://img.jgi.doe.gov/genome.php?id=2802428848) | 3.5 | 339 | 90 | 2 | 56.9 |
