# Supplementary material for: Woeseiales transcriptional response to shallow burial in Arctic fjord surface sediment
Source: PLoS One. 2020 Aug 27;15(8):e0234839. doi: 10.1371/journal.pone.0234839 (PMC7451513; doi:10.1371/journal.pone.0234839)
Supplement: S2 Table — C = Energy production and conversion; D = Cell cycle control, cell division, chromosome partitioning; E = Amino acid transport and metabolism; F = Nucleotide transport and metabolism; G = Carbohydrate transport and metabolism; H = Coenzyme transport and metabolism; I = Lipid transport and metabolism; J = Translation, ribosomal structure and biogenesis; K = Transcription; L = Replication, recombination and repair; N = Cell motility; O = Post-translational modification, protein turnover and chaperones; P = Inorganic ion transport and metabolism; Q = Secondary metabolites biosynthesis, transport and catabolism; S = Function unknown; T = Signal transduction mechanisms; U = Intracellular trafficking, secretion and vesicular transport; V = Defense mechanisms. (DOCX) [file pone.0234839.s003.docx]

S2 Table. Genes with transcripts detected at all sites. **C** = Energy production and conversion;

**D** = Cell cycle control, cell division, chromosome partitioning; **E** = Amino acid transport and metabolism; **F** = Nucleotide transport and metabolism; **G** = Carbohydrate transport and metabolism; **H** = Coenzyme transport and metabolism; **I** = Lipid transport and metabolism; **J** = Translation, ribosomal structure and biogenesis; **K** = Transcription; **L** = Replication, recombination and repair; **N** = Cell motility; **O** = Post-translational modification, protein turnover and chaperones; **P** = Inorganic ion transport and metabolism; **Q** = Secondary metabolites biosynthesis, transport and catabolism; **S** = Function unknown; **T** = Signal transduction mechanisms; **U** = Intracellular trafficking, secretion and vesicular transport; **V** = Defense mechanisms.

| Gene Product | COG |
| --- | --- |
| 2-oxoglutarate oxidoreductase subunit KorB | C |
| 6-hydroxy-D-nicotine oxidase | C |
| ATP synthase subunit alpha | C |
| ATP synthase subunit beta | C |
| Citrate synthase | C |
| CoB--CoM heterodisulfide reductase iron-sulfur subunit D | C |
| Cytochrome subunit of sulfide dehydrogenase | C |
| Hdr-like menaquinol oxidoreductase iron-sulfur subunit 2 | C |
| Hydrogenase-1 large chain | C |
| Isocitrate lyase | C |
| NAD(P) transhydrogenase subunit beta | C |
| Nitrite reductase | C |
| Pyruvate synthase subunit PorA | C |
| Sulfide dehydrogenase subunit alpha | C |
| Sulfite reductase, dissimilatory-type subunit beta | C |
| Uptake hydrogenase small subunit | C |
| Cell division protein FtsZ | D |
| Chromosome-partitioning ATPase Soj | D |
| Iron-sulfur cluster carrier protein | D |
| 2,5-dioxopentanoate dehydrogenase | E |
| Dipeptidyl carboxypeptidase | E |
| NAD-specific glutamate dehydrogenase | E |
| NADPH-Fe(3+) oxidoreductase subunit beta | E |
| Carbamoyl-phosphate synthase large chain | F |
| Inosine-5'-monophosphate dehydrogenase | F |
| Uridylate kinase | F |
| Vitamin B12-dependent ribonucleoside-diphosphate reductase | F |
| Monocarboxylate 2-oxoacid-binding periplasmic protein | G |
| Adenosylhomocysteinase | H |
| 3-oxoacyl-[acyl-carrier-protein] reductase FabG | I |
| Acetyl-coenzyme A synthetase | I |
| Acyl-CoA dehydrogenase | I |
| 30S ribosomal protein S12 | J |
| 30S ribosomal protein S17 | J |
| 30S ribosomal protein S5 | J |
| 50S ribosomal protein L1 | J |
| 50S ribosomal protein L10 | J |
| 50S ribosomal protein L18 | J |
| 50S ribosomal protein L2 | J |
| 50S ribosomal protein L21 | J |
| 50S ribosomal protein L25 | J |
| 50S ribosomal protein L7/L12 | J |
| Elongation factor Tu | J |
| Glutamine--tRNA ligase | J |
| Phenylalanine--tRNA ligase beta subunit | J |
| Polyribonucleotide nucleotidyltransferase | J |
| tRNA pseudouridine synthase D | J |
| Cold shock-like protein CspE | K |
| DNA-directed RNA polymerase subunit beta | K |
| DNA-directed RNA polymerase subunit beta' | K |
| ECF RNA polymerase sigma factor SigW | K |
| HTH-type transcriptional repressor BluR | K |
| Integration host factor subunit alpha | K |
| Phage shock protein A | K |
| RNA polymerase sigma factor RpoD | K |
| RNA polymerase-binding transcription factor DksA | K |
| DNA topoisomerase 1 | L |
| DNA-binding protein HU | L |
| Twitching mobility protein | N |
| Type II secretion system protein E | N |
| 60 kDa chaperonin | O |
| ATP-dependent zinc metalloprotease FtsH | O |
| Chaperone protein ClpB | O |
| Chaperone protein DnaK | O |
| Chaperone protein HtpG | O |
| Glutathione amide-dependent peroxidase | O |
| Lon protease | O |
| Membrane-bound protease | O |
| Modulator of FtsH protease HflK | O |
| Neutral endopeptidase | O |
| Spore protein SP21 | O |
| Stomatin | O |
| Arylsulfatase | P |
| Catalase-peroxidase 2 | P |
| Colicin I receptor | P |
| Hemin receptor | P |
| Vitamin B12 transporter BtuB | P |
| Dehydrosqualene desaturase | Q |
| Cation/acetate symporter ActP | S |
| Metalloprotease PmbA | S |
| Nitrilase | S |
| Aerobic respiration control sensor protein ArcB | T |
| Biopolymer transport protein ExbB | U |
| Fimbrial protein | U |
| Protein translocase subunit SecY | U |
| Type II secretion system protein F | U |
| Type IV pilus biogenesis and competence protein PilQ | U |
| Macrolide export ATP-binding/permease protein MacB | V |
