# Supplementary figures and images for: Woeseiales transcriptional response to shallow burial in Arctic fjord surface sediment
Source: PLoS One. 2020 Aug 27;15(8):e0234839. doi: 10.1371/journal.pone.0234839 (PMC7451513; doi:10.1371/journal.pone.0234839)

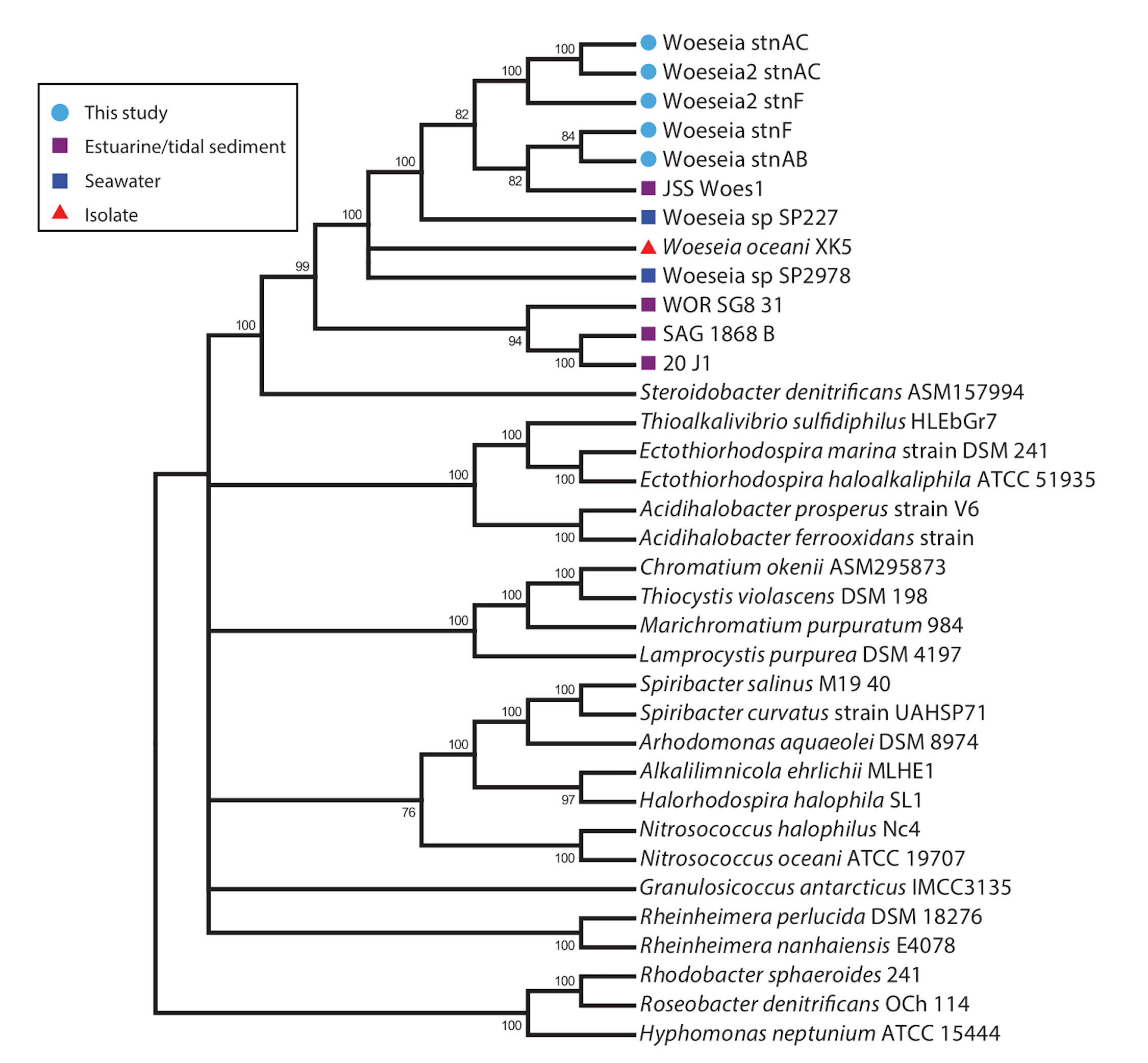

Supplement: S1 Fig — Maximum likelihood was calculated in Mega v. 7 with 1000 bootstraps on a concatenated alignment of 49 ribosomal proteins. Only nodes with >75% support are shown. MAGs from this study are indicated with blue circles. Tree was rooted using Roseobacter species as the outgroup. (TIF) [file pone.0234839.s004.tif]

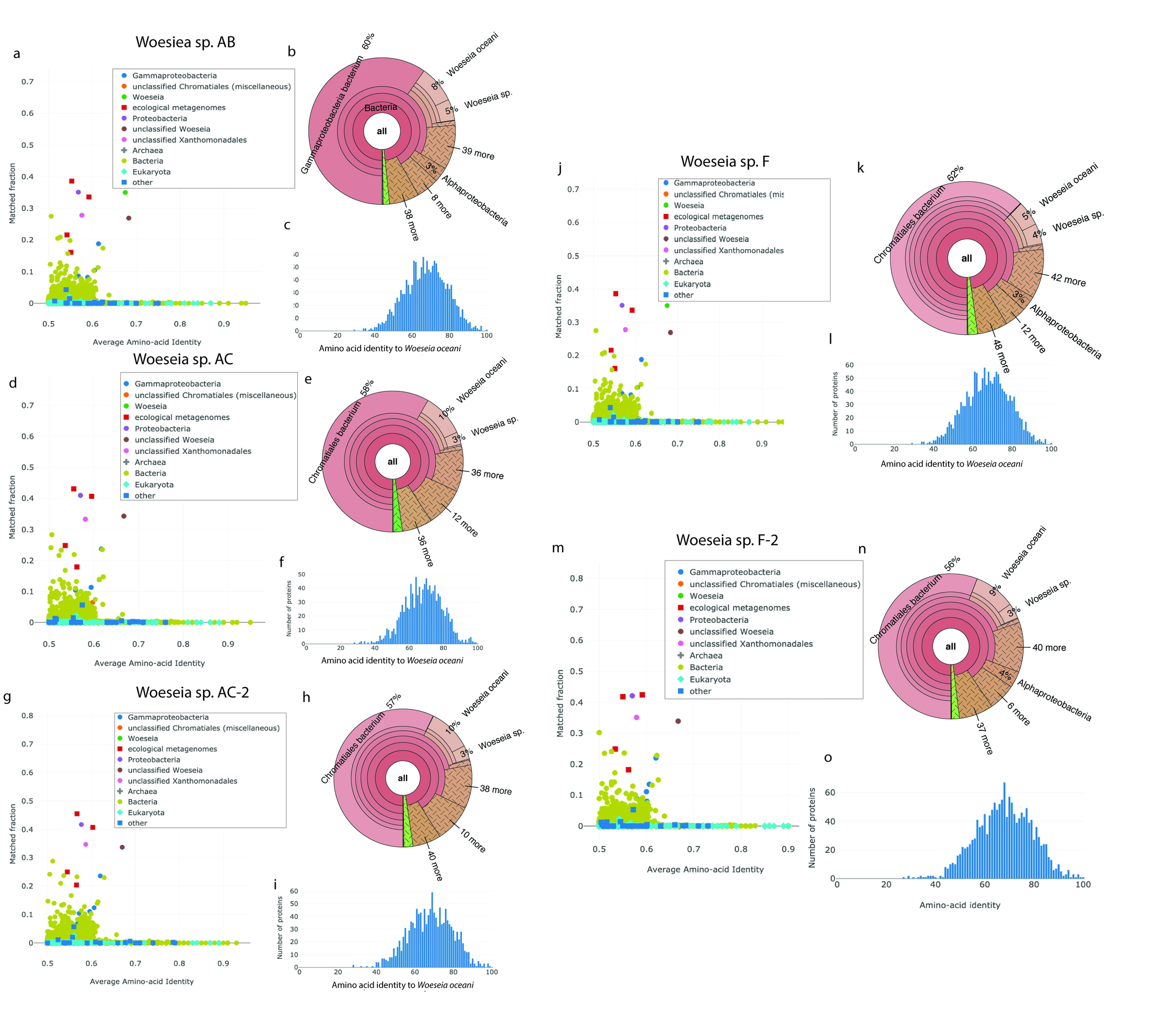

Supplement: S2 Fig — Figure generated with KEGG-decoder [26]. (TIF) [file pone.0234839.s005.tif]

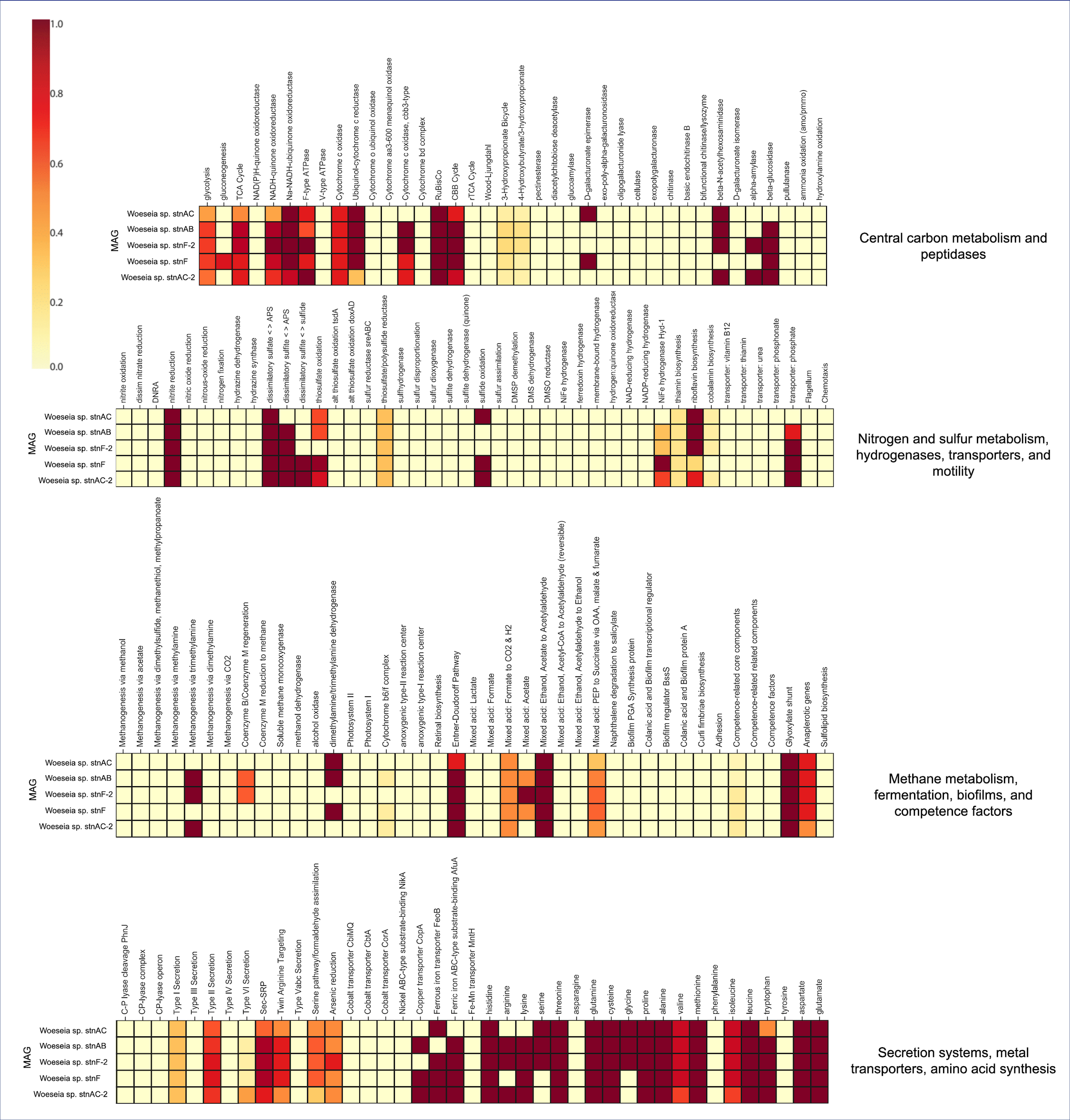

Supplement: S3 Fig — Scatterplots, Krona diagrams, and histograms relating amino acid identity to the cultured isolate, Woeseia oceani, are presented for the MAGs Woeseia_stnAB (a, b, c), Woeseia_stnAC (d, e, f), Woeseia2_stnAC (g, h, i), Woeseia_stnF (j, k, l), and Woeseia2_stnF (m, n, o). (TIF) [file pone.0234839.s006.tif]

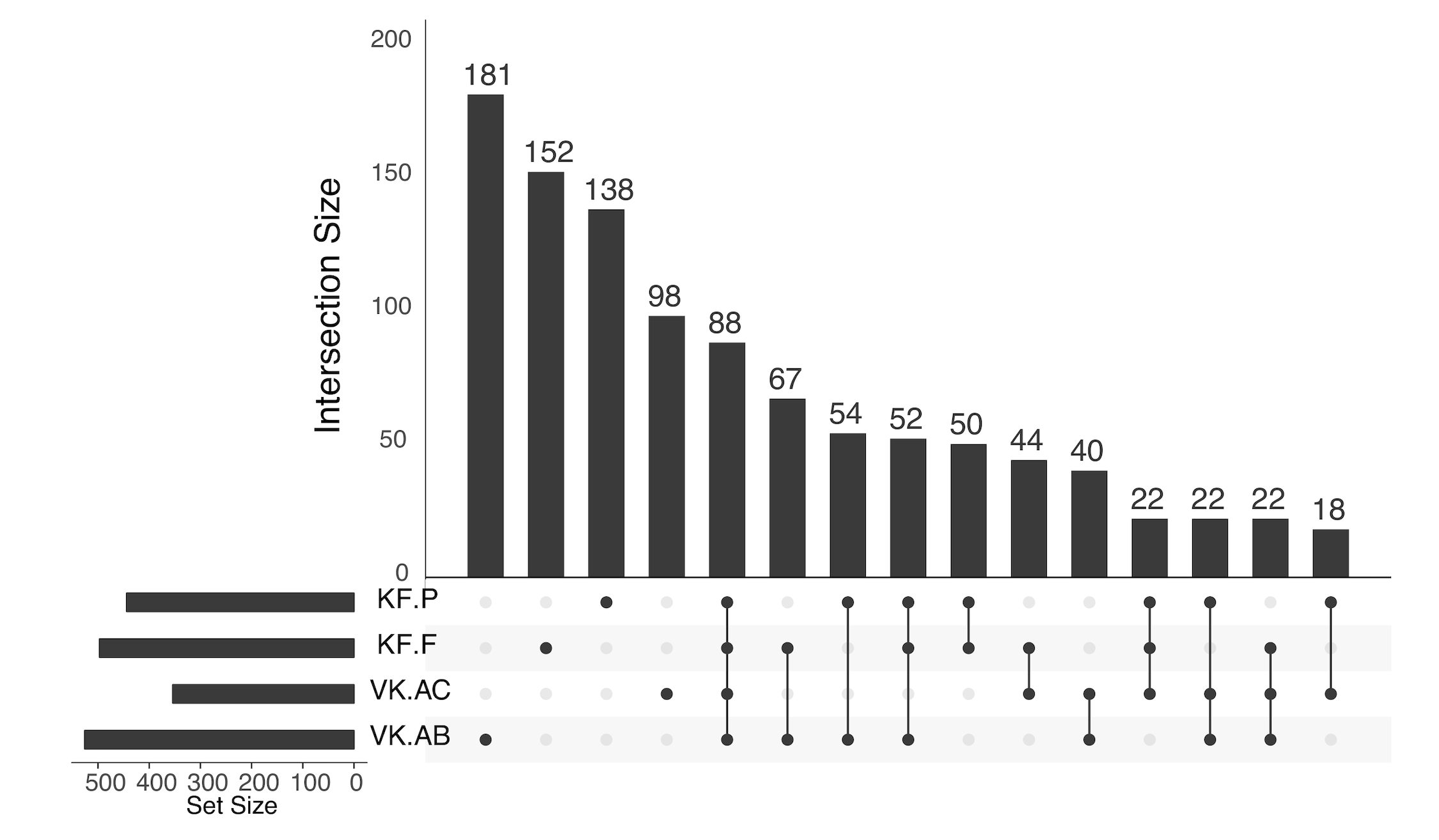

Supplement: S4 Fig — Set intersections of transcripts visualized with UpsetR [86]. The total number transcribed genes (“set size”) is indicated as horizontal bars. Vertical bars represent the intersection size, or number of transcribed genes that fall into the 15 different sets of site combinations. Depth analysis of transcript abundance for unique sets of transcripts are depicted in S5 Fig. (TIF) [file pone.0234839.s007.tif]

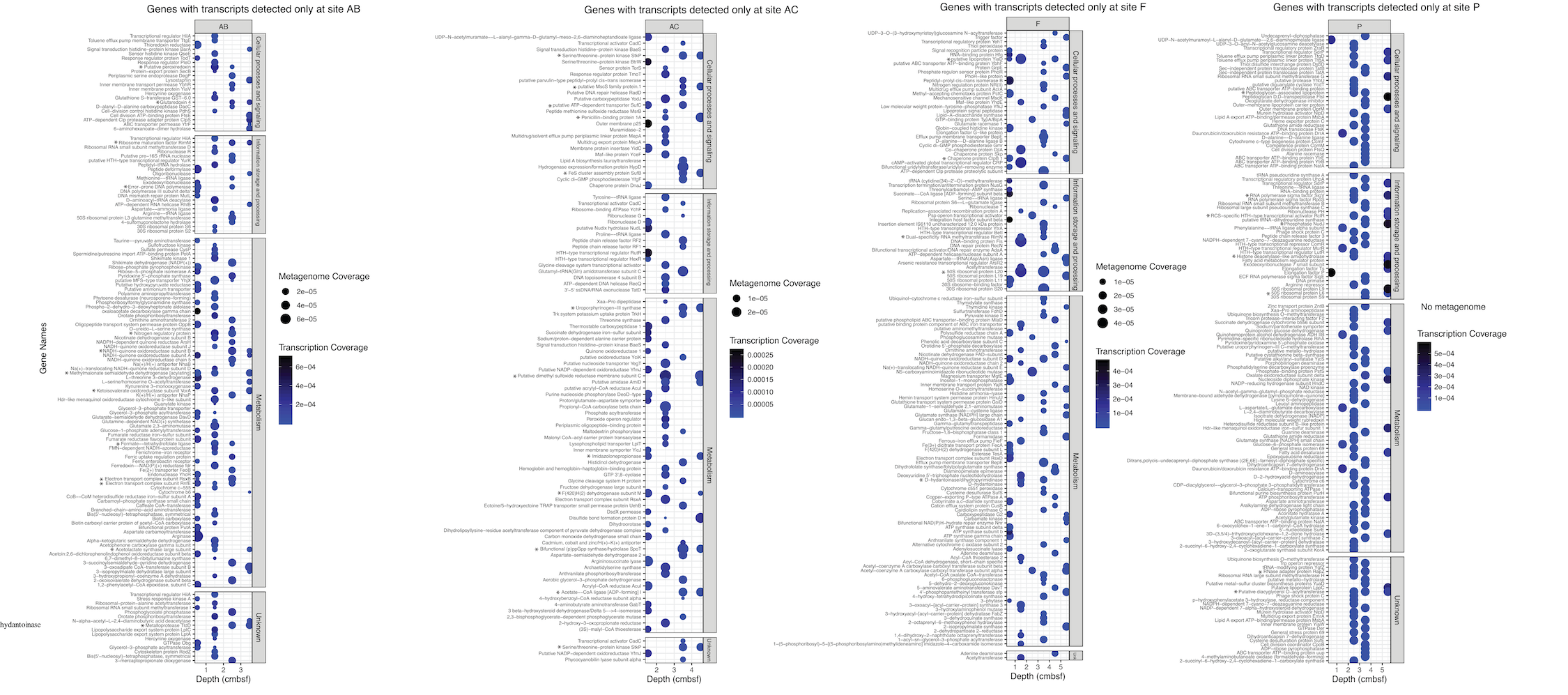

Supplement: S5 Fig — Transcriptional coverage (transcript abundance normalized to gene length) is reported for the unique transcripts detected at each site for the COGs Cellular Processing and Signaling, Information Storage and Processing, Metabolism, and Function Unknown. Those with predicted function only were removed from this analysis. Dot size scales with metagenome coverage and color saturation scales from lighter to darker blue for increased transcriptional coverage. Site P did not have a metagenome. (TIF) [file pone.0234839.s008.tif]

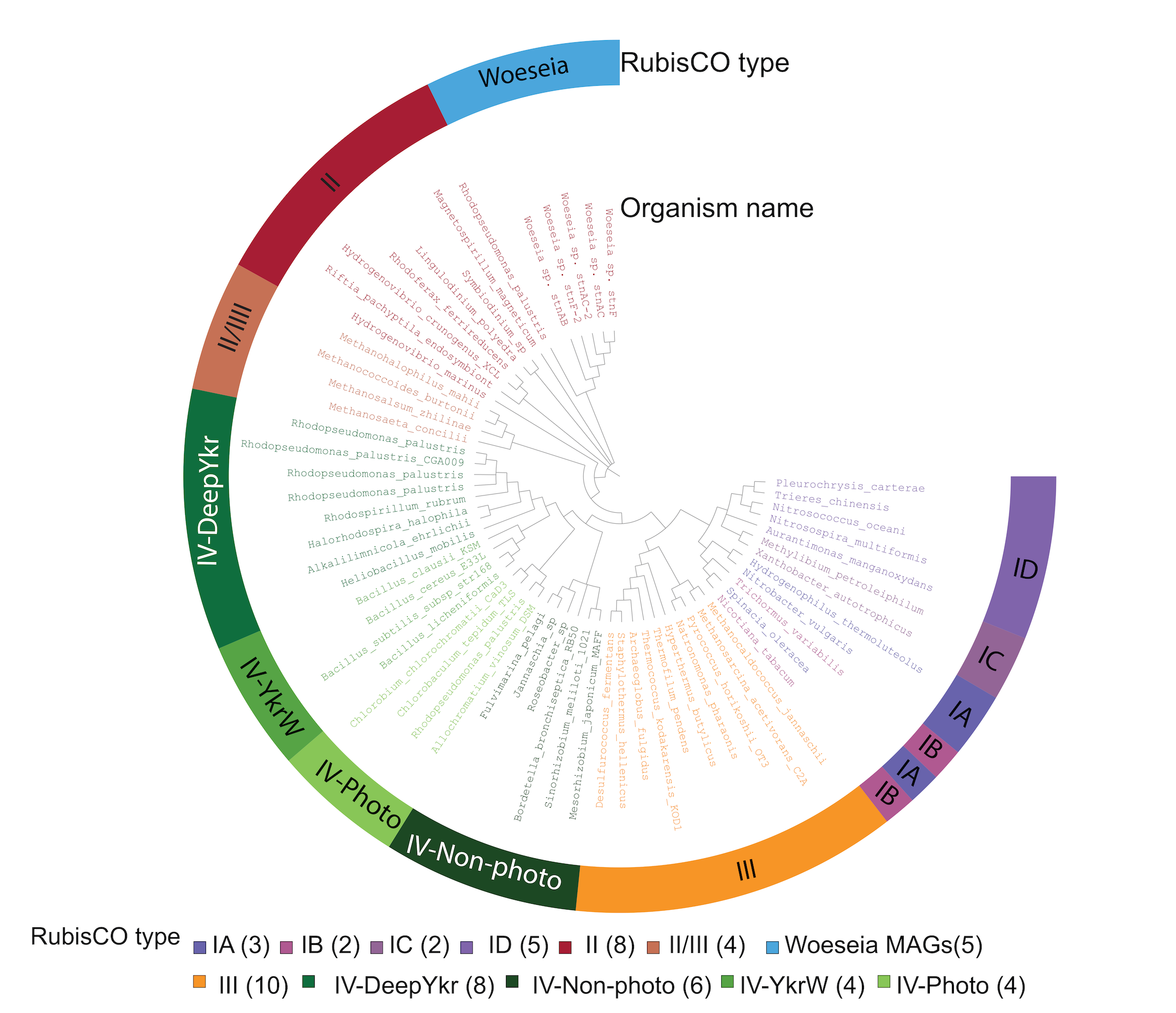

Supplement: S6 Fig — Only nodes with >80% support after 1000 bootstraps are shown. (TIF) [file pone.0234839.s009.tif]

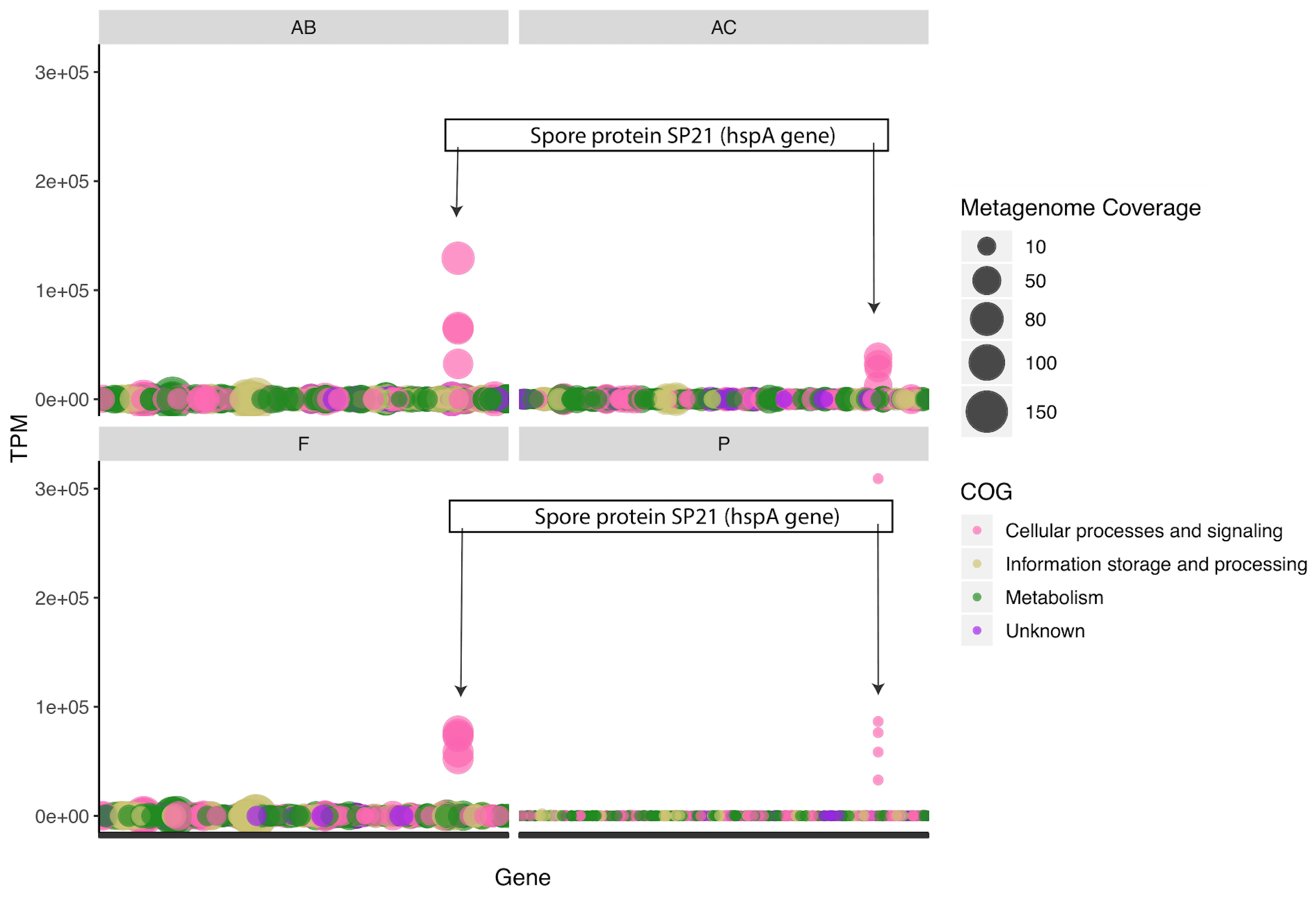

Supplement: S7 Fig — The transcripts per million (TPM) value is reported for each transcribed gene (along x-axis). Names of genes are not included to ease illustration and interpretation. COG assignment of genes is represented by color. The gene encoding for spore protein SP21 is indicated with arrows. (TIF) [file pone.0234839.s010.tif]

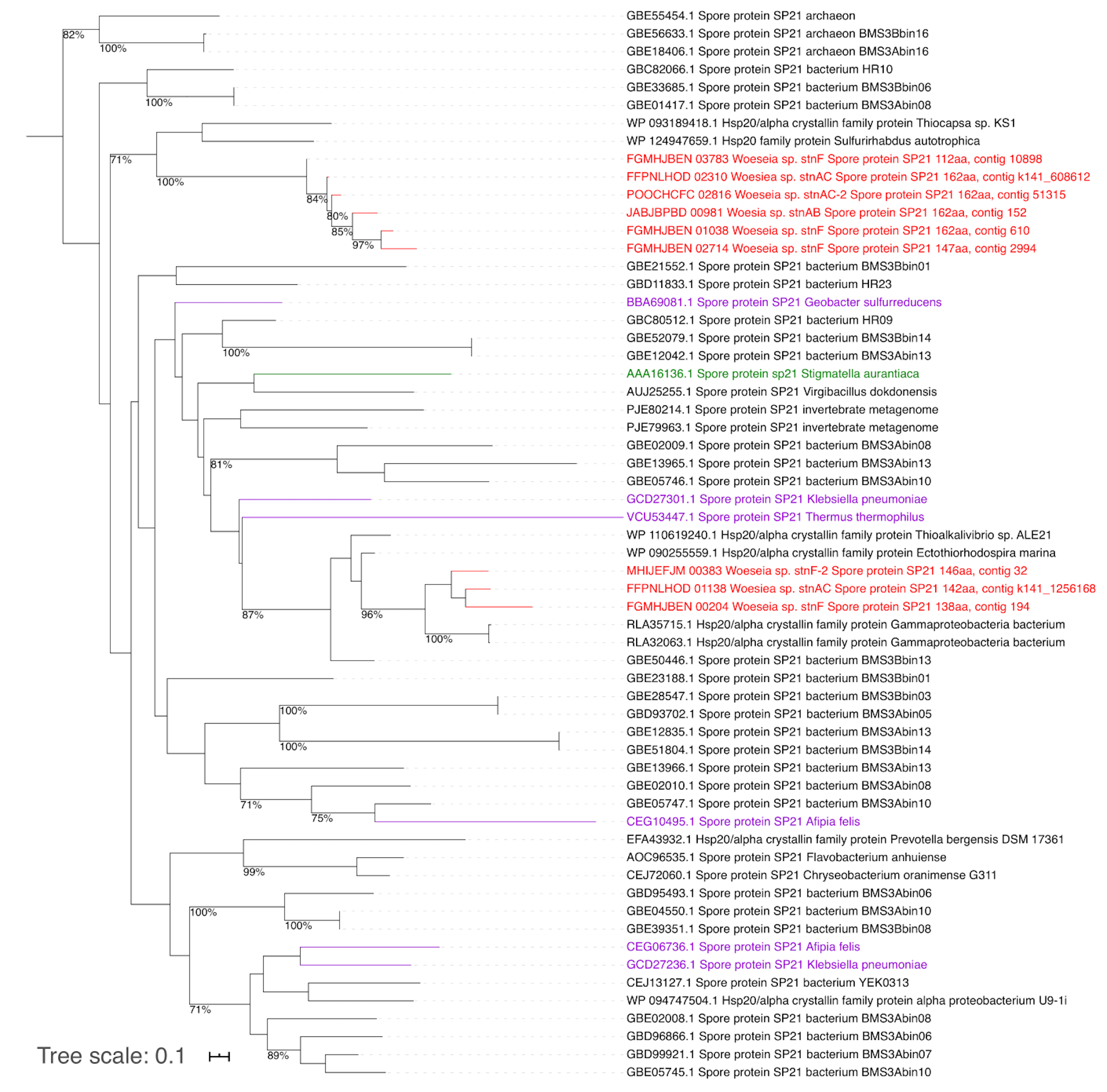

Supplement: S8 Fig — Bootstrap support after 1000 bootstraps is reported for nodes with > 70% support. Woeseiales sequences are indicated in red, cultured microorganisms in purple, and experimentally-verified spore protein SP21 indicated in green [36, 45]. Archaeal sequences of spore protein SP21 were used as the outgroup. The scale bar represents the number of substitutions per site. (TIF) [file pone.0234839.s011.tif]

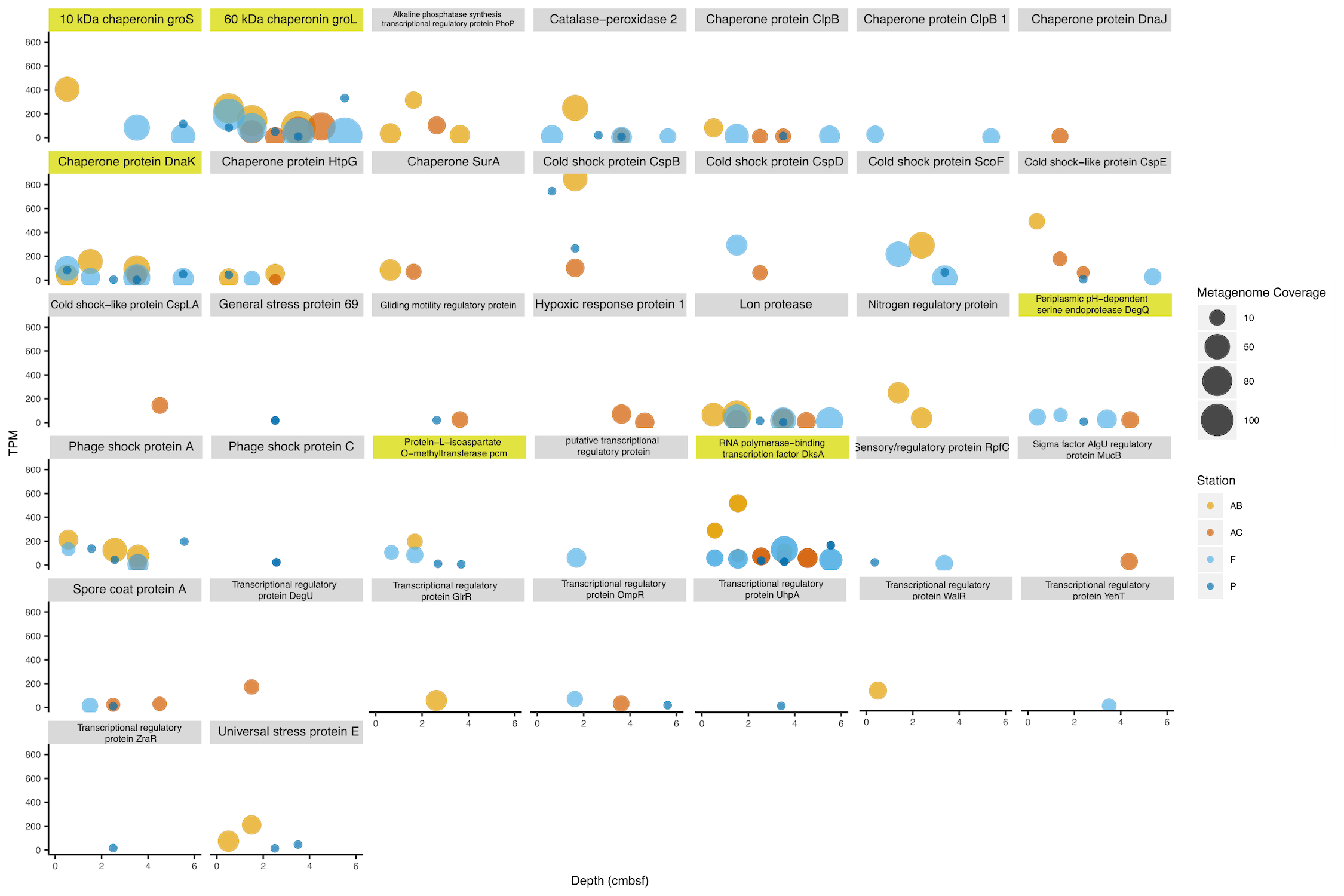

Supplement: S9 Fig — TPM values reported for genes associated with starvation response, stress mitigation, protein repair/folding, and transcriptional regulation. Yellow border labels indicate genes that co-occur on the same contigs as hspA (encoding spore protein SP21; S9 Fig). (TIF) [file pone.0234839.s012.tif]

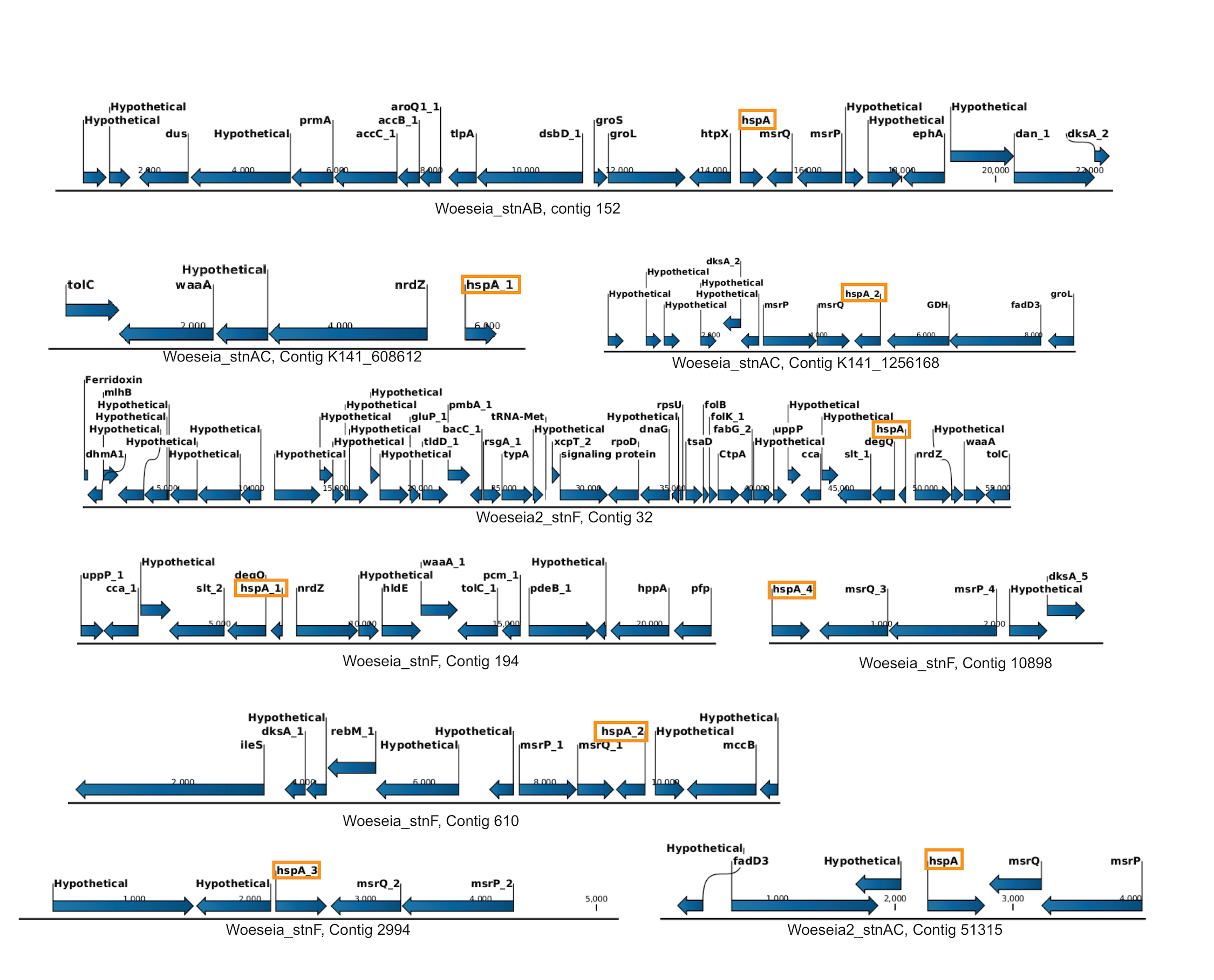

Supplement: S10 Fig — Visualizations from CLC Genomics Workbench. (TIF) [file pone.0234839.s013.tif]
